# Systematic Overview of Solid Particles and Their Host Responses

**DOI:** 10.3389/fimmu.2018.01157

**Published:** 2018-05-28

**Authors:** Fei Shu, Yan Shi

**Affiliations:** ^1^Department of Basic Medical Sciences, Institute for Immunology, Center for Life Sciences, Beijing Key Laboratory for Immunological Research on Chronic Diseases, Tsinghua University, Beijing, China; ^2^Peking University-Tsinghua University-National Institute of Biological Sciences Joint Graduate Program, School of Life Sciences, Peking University, Beijing, China; ^3^Department of Microbiology, Immunology and Infectious Diseases, Snyder Institute, University of Calgary, Calgary, AB, Canada

**Keywords:** solid particle, host response, biological crystal, adjuvant, cell death

## Abstract

Crystalline/particulate substances trigger a plethora of signaling events in host cells. The most prominent consequence is the inflammatory reactions that underlie crystal arthropathies, such as gout and pseudogout. However, their impact on our health was underestimated. Recent work on the role of cholesterol crystal in the development of atherosclerosis and the harm of environmental particulates has set up new frontiers in our defense against their detrimental effects. On the other hand, in the last 100 years, crystalline/particulate substances have been used with increasing frequencies in our daily lives as a part of new industrial manufacturing and engineering. Importantly, they have become a tool in modern medicine, used as vaccine adjuvants and drug delivery vehicles. Their biological effects are also being dissected in great detail, particularly with regard to their inflammatory signaling pathways. Solid structure interaction with host cells is far from being uniform, with outcomes dependent on cell types and chemical/physical properties of the particles involved. In this review, we offer a systematic and broad outlook of this landscape and a sage analysis of the complex nature of this topic.

## Introduction

Solid amorphous/crystalline/fibrous particles are common in our environment. Looking outward, all life forms are exposed to particles varying in size, chemistry, and the state of agglomeration in the surroundings. With the technological advancements, human populations are facing new variations related to the exposure to environmental and/or occupational pollutants/hazards/toxins. In recent times, nanotechnologies bring ultrafine particles into our lives (1). Therefore, we are in an ever-changing world of particulate substances. Looking inward, several essential biological components are in delicate balance near the point of precipitation, including nucleic acid metabolites, lipids, and ions. Some forms of solidification, such as cholesterol crystal (CC) formation and calcification of joints, are a part of aging physiology. Other precipitations, such as monosodium urate (MSU) (2), can lead to acute inflammation and tissue damage. In addition, with advancements in modern medicine, pharma-biotech companies/institutes introduce particles in formulation development for vaccination, drug delivery and cancer therapy. Compared with other areas of biomedical research, so far there has not been a discipline dedicated to studying how hosts respond to solid structures. As such, our understanding and theories are mostly fragmented, creating a hidden deficit in our effort to control and utilize this class of materials.

Host responses to solid/crystalline particles have been studied by scientists and clinicians for decades for health concerns. Historical lessons are vivid. One of the most memorable is the chrysotile and amphibole asbestos-induced respiratory deficiencies and mesothelioma (3, 4). The impact was that guidelines were imposed by regulatory bodies to strictly ban its consumer use and minimize it human exposure of occupational operators (https://www.epa.gov/asbestos). In recent years, cellular signaling events associated with crystal-triggered inflammation have become an intensely investigated topic (5). Some areas are covered with extreme depths, including inflammasome activation (6), cell death (7–9), reactive oxygen species (ROS) production (10, 11), and adjuvanticity (12, 13). However, crystals vary greatly in their geometry and chemistry. Host cells with whom they interact are also diverse. Therefore, the speedy accumulation of newly gained knowledge has not led to a sufficient number of consensuses. In addition, a solid structure/host cell response cannot be comprehended at any given cross section. It is a chain of events from crystal formation/entry, cell membrane binding, intracellular signaling cascades, cytokine release, cell death, secondary host responses, etc. In this review, we aim to integrate several less illuminated areas of cell responses to solid particles, including physiochemical properties, nanoparticles, particulate adjuvants, and ongoing debates regarding their activation mechanisms. In other words, we offer a panoramic view of this interesting topic.

## Physical and Chemical Properties

While research on signaling events in host response to solid particles is currently very active, the study of their chemical and physical properties remains a widely pursued subject. As particles under analysis and experimental readouts vary, it is hard to draw a set of conclusions with any precision. The overarching observations are: (1) Size of any given solid structure has a great impact on its biological effects. (2) A given volume of a particulate substance can have different effects depending on their geometric parameters. (3) Surface chemistry, including coating, can change outcomes. (4) Different host cell types can manifest different host responses. However, if the nature and types of experimental outcomes can hypothetically be set aside, and only activation intensity (i.e., cytokine production, adjuvanticity, and cellular morphology changes) is used as the readout, some internal consistencies can be extracted from the existing literature.

### Size

Generally, solid structures of size 1–100 nm are considered as nanoparticles. For these particles, the general entry into the cells is mediated by endocytosis or simple diffusion without any defined receptors. Sizes beyond 100 nm and up to 10 µm are regarded as microcrystals. Within the latter, for particles with a diameter larger than 0.5 µm, phagocytosis is the dominant form of entry (14). There have been some isolated studies (15–17) whereby the size of polylactide-co-glycolide (110 vs 800–900 nm) did not show any significant changes in the ability to induce antibody responses to protein antigens from *Neisseria meningitidis* and HIV in mice (18). However, most papers appear to offer different results (1). Early work using simple stimulation and cell proliferation index measurement suggested that larger surface area (smaller diameter) of polystyrene and TiO_2_ particles seemed to block macrophage cell line proliferation *in vitro* (taken as an indicator of cell loss) (19). This finding was confirmed by another group where carbon black as well as TiO_2_ particles were used *in vivo*. In that study, both particles of 250 nm in diameter and those roughly one log smaller were instilled intratracheally. In rats, the smaller ultrafine particles induced drastically higher neutrophil infiltration in the lung and greater epithelial damage (20). Using epithelial cells as an example, amounts (total volume) required for small (below 20 nm) vs large (above 100 nm) silica crystals tended to create a log difference in the efficiency of inducing cell death. In addition, smaller particles achieved the same effect much faster than the larger ones in human endothelial cells *in vitro* (21). Similar observations were made by other groups studying human endothelial cells and macrophage *in vitro* (22). One reason for this difference is that small particles enter cells more readily. Using silver nanoparticles (SNP) SNP-5, SNP-20, and SNP-50 (numbers indicate diameter in nanometer) as an example, *in vitro* cellular toxicity of smaller particles was correlated with their rapid presence inside the human epithelial cells (23). In one report that compared nano vs micro silica particles, smaller (30–1000 nm) crystals entered mouse BMDM easily and caused significant lysosomal marker loss, indicating lysosome destabilization, in comparison with the larger ones (1,000–10,000 nm) (24). An interesting contrast was another paper suggesting that silica particles 1,000 nm across were more toxic than small (30 nm) to THP-1 cells (25), a phenomenon associated with the efficient uptake of the larger particles. This study, as well as several others, suggested that phagocytes, such as J774.2 and RAW264.7 cells, were more prone to particle-induced cell damage than nonphagocytic cells (L929) (26). These results imply that efficient entry may ultimately explain the ability to trigger cellular responses. Whether additional signaling mechanisms related to particle size also account for the stimulation intensity have not been independently investigated.

### Morphology and Geometry

Shape of solid structures has been implicated in some studies to be a critical factor in triggering host cell responses. The differences in crystalline symmetry, i.e., anatase vs rutile TiO_2_, could result in significantly different outcomes in mouse keratinocytes *in vitro* (27). The sharp and pointy edges of many crystals, i.e., asbestos and MSU, are believed to at least partially contribute to their pathology *via* direct injury to mouse mesothelial cell membrane *in vitro* and *in vivo* (28, 29). Using non-opsonized hydroxyapatite (HA) as an example, a study was conducted to compare four types of geometries: needle, plate, sphere, and rod and tested their ability to induce TNFα/IL-6 and ROS production as well as cytotoxicity. It was found that needle and plate shapes induced the highest rate of cell death in human bronchial epithelial cells accompanied by high IL-6 production. Interestingly, rod-shaped HA induced more ROS production. RAW264.7 cells, on the other hand, showed much less selectivity to the shape in all the parameters measured (30). A study on carbon nanotubes (CNTs) also suggested that long and needle-shaped CNTs and asbestos triggered human macrophage IL-1β secretion *in vitro* while only the former triggered IL-1α production. Carbon black and short CNTs failed to induce either (31). Interestingly, in this report, it was found that long CNTs induced a typical NACHT, LRR, PYD domains-containing protein 3 (NLRP3) inflammasome activation event that relied on ROS production, P2X7 receptor, and lysosomal destabilization. Long silver nanowires were also more inflammatory toward human epithelial and liver cells *in vitro* than the short ones (23), and spherical TiO_2_ was less stimulatory than the same material in the shape of nano belts *in vivo* (32). The observations may be associated with higher area/volume ratio, although a systematic analysis is not yet available. In a study comparing crystalline nanocellulose with fibrillary cellulose, it was found in A549 cells fibrillary cellulose was more toxic than the crystalline counterpart. This was mainly due to the former’s strong ability to induce oxidative stress. On the other hand, crystalline cellulose was able to induce a broad range of cytokine production including IL-6, IL-8, MCP-1, IL-12p70, and G-CSF (33). Therefore, distinct shapes with similar chemistry can lead to different profiles of cellular responses. Many crystalline structures may also exist in an amorphous state by contrast to the better known larger crystals. In a pulmonary inflammation mouse model, amorphous/colloidal silica induced only transient inflammation while the response triggered by crystals was more persistent (34). Indeed, for non-crystalline structures such as SiO_2_ from nano to micro μm sizes (mono-disperse and poly-disperse), the stimulation for NLRP3 inflammasome activation in mouse macrophages was lower than the crystalline. On the other hand, the comparison between amorphous and colloidal forms did not reveal any difference in stimulation capacity (35).

### Chemical Composition

Chemical properties of solid structures have been directly linked to their stimulatory ability. Under this umbrella, one consideration is the degree of solubility. It is generally considered that solubility is proportionally related to cytotoxicity (36). In several reports, slightly soluble zinc and iron oxides were more potent in inducing target cell DNA release than almost insoluble CeO_2_, ZrO_2_, TiO_2_, and Ca_3_(PO_4_)_2_. In one study, ionic metal was critical to IL-6 and IL-8 production by human airway epithelial cells which were blocked by metal chelation (37). However, mechanistic insights of the observations of this nature are not available (38, 39). In the absence of detectable solubility, chemical composition can also make a difference in cellular responses. By comparing various metal oxides, copper oxide had the highest toxicity toward airway epithelial cells (40). In that study, several metal oxides were equally potent to induce ROS in the treated cells, however, CuO had the ability to suppress the cellular antixidation effects, i.e., the activities of catalase and glutathione reductase. It should be noted that depending on cell types studied and experimental settings, results have not been consistent regarding chemical compositions of particles. In some studies, Y_2_O_3_ (yttrium oxide) and ZnO were found to trigger inflammatory responses (ICAM-1, MCP-1, and IL-8 expression) better than Fe_2_O_3_ in endothelial cells (41), by contrast with the report by Brunner et al. where iron oxides were more stimulatory in human mesothelioma and rodent fibroblast (36). Surface charge and hydrophobicity may also affect cellular responses *in vivo* (42–44). This notion was echoed by a study where unmodified silica crystals induced strong IL-1β, ROS, and NLRP3 inflammasome activation in THP-1 cells. However, surface functionalization with –COOH, –NH_2_, –SO_3_H, and –CHO groups significantly reduced all aspects of inflammatory responses (45). In fact, the simple presence of cell culture serum can result in significant reduction of toxicity toward fibroblast, which presumably was driven by the alteration of particle surface chemistry (46). Interestingly, the different surface chemistry can also alter their anatomic distributions. For instance, i.p. injected polymethyl methacrylate beads tended to deposit in the spleen resulting in its enlargement. Polystyrene beads of similar sizes, on the other hand, were accumulated in adipose tissues (47). Likely the different surface chemistries triggered different migration patterns in the phagocytosing macrophages.

It should be noted that the forth mentioned experimental results are small samples of the vast literature on chemical/geometric properties of host-interacting particles. Depending on cell types, readouts, and experimental settings, different and even contradictory reports are common. Ideally, the desired approach is to isolate one particular variable for extensive analysis while other parameters are meticulously controlled. Thus far, the boldest attempt to extract a set of principles underneath the surface chemistry and immune recognition was made by Williams et al. (48). In that study, they used “layered double hydroxides” (LDH) for analysis, prompted by the effect of alum in immune stimulation. The exact chemical compositions are technically challenging to understand. We can picture their setup as follows. A sheet of metal (M^+^M^2+^ and M^3+^) hydroxides is laid against pairing anions to form a stack. Each stack is laid on top of another for a multilayered structure. Because the metal ions can be chosen, M^+^M^2+^ vs M^3+^ ratios and anion species can be selected, the resulting structures can be tested for their immune regulation solely as a function of various ions used in the experiment. Dendritic cell (DC) activation was measured by a set of cytokine production. LiAl_2_-CO_3_, Mg_2_Al-NO_3_, Mg_2_Fe-Cl, Imject alum, and alhydrogel were compared. Surprisingly, this study revealed that all *in vitro* human DC responses were highly correlated with a linear combination of three LDH properties: the radius of the spherical M^+^ or M^2+^ metal cations; the distance between the LDH layers (interlayer spacing); and zeta potential that defines the magnitude of the electrical charge at the interfacial double layer around the LDH particle. Newly synthesized LDHs were highly predictable by these variables in their DC stimulatory capacity. These properties were directly verified in *in vivo* mouse antibody production. These efforts were aimed to produce a set of “chemical–immunology rules”. Clearly, to understand the complex nature of host responses to solid structures, undertakings like this point to a possible angle to tackle the vast unknowns of physics and chemistry of particulate substances involved in host cell activation.

## Biological Crystals

Unlike particles that come with modern manufacturing/processing or exist in our environment, crystalline deposition has long been a part of human biology/pathology. Best known among them are uric acid, cholesterol, heme, and a list of calcium-containing crystals [calcium pyrophosphate dihydrate (CPPD), HA, calcium oxalate, and calcium phosphate family in general]. Slightly less prevalent diseases can be caused by additional crystals, such as xanthine leading to xanthinuria and arthropathy (49) and cystine (oxidized cysteine dimer) in kidney stones (50). A rare genetic disease, adenine phosphoribosyltransferase deficiency, results in the inability to produce adenosine monophosphate from adenine, 2,8-dihydroxyadenine crystal formation, and kidney failure in human and mice (51–54). In this section, we aim to illustrate the most common types and their related pathologies.

### Monosodium Urate Crystals

Gout, the deposition of MSU crystals, has been recognized for over 4,000 years and extensively described in the ancient literature (55). Historically, its occurrence has been associated with excessive dietary and alcohol consumption (56). In the 18^th^ century, crystals from a tophaceous joint were isolated and their chemical nature was reported (55). It is generally believed that high purine metabolism leads to hyperuricemia (>6.8 mg/dl), a precondition for gout and tophus. Gout is often induced by metabolic and environmental factors, such as increased Ca^2+^, low pH, and cold weather, and occurs only in the distal extremities, never near the core of body where the temperature is more consistent (57). Interestingly, till date, we still cannot duplicate the *in vivo* crystal formation event in the lab, as at this concentration uric acid does not precipitate in standard buffers (58). Additional factors, for instance natural MSU antibodies, may be critical for this process (58, 59). Mechanistically, *in vivo* the initial nucleation of uric acid crystals is reversed due to rapid dissolution. The antibodies (IgG in human gout patients and IgM in mouse) help stabilize the nucleation core whereby the crystal growth is permitted. This notion is supported by clinical observations that MSU crystals isolated from patients are often coated with a layer of antibodies (60), with Fab pointing to the crystalline surface (61). Regarding the signaling events leading to the painful inflammatory episode of gouty arthritis, many models have been proposed. MSU typically activates NLRP3 inflammasome and IL-1β production, and the proposed signaling events pertinent to NLRP3 regulation, such as ROS, K^+^ efflux, and lysosomal rupture, are all implicated in its inflammatory properties (discussion later). Specific to MSU, it has been suggested that CD11b and CD16 may directly recognize MSU crystals because antibodies for these two surface molecules reduced MSU-mediated neutrophil activation (62, 63). Interestingly, the antibody blockage also reduced neutrophil responses to CPPD, a chemically distinct structure, suggesting that these surface molecules may merely participate in the signaling rather than the specificity determinant for these crystalline surfaces. Liu-Bryan and Terkeltaub’s group reported that toll-like receptor (TLR)2/TLR4 and CD14 were the functional receptors of MSU (64–66). The conclusion was mostly drawn from the reduced inflammatory responses in mice deficient in these genes. Using similar TLR-deficient mouse models as well as *in vitro* cell transfection, Chen et al. failed to see any involvement of TLR2 or TLR4 (67). While the suggestion of protein-based positive signaling receptor for MSU has not been further investigated, one paper suggested that Clec12a is an inhibitory receptor for this crystal. Binding analysis showed that Clec12a had specific affinity for MSU and mice deficient in this gene mounted increased inflammatory responses against MSU challenge (68). Our lab’s results have suggested another model. The surface of MSU crystal showed substantial binding to cholesterol, a component of lipid rafts. The binding event caused the plasma membrane lipid sorting and an accumulation of ITAM-containing membrane proteins. This accumulation, in turn, recruited Syk to the inner leaflet and induced a chain reaction similar to Syk/PI3K-dependent phagocytic activation (69). This model describes a lipid-based signaling event independent of protein receptors. Whether this signaling modality is central to the general sensing of solid structures and is being actively investigated.

### Calcium Crystals

While calcium salt crystals mediate inflammatory responses similar to MSU (70), the prerequisites for their formation are different. First, calcium-containing crystal generation does not require elevated levels of Ca^2+^. Second, these crystals almost always develop on matrix surfaces, mostly cartilages (71). Inorganic pyrophosphate, produced *via* ATP metabolism, is found to induce CPPD formation. On cartilages, inorganic pyrophosphate level is regulated by several enzymatic activities including ectonucleotide pyrophosphatase (72, 73). Unlike MSU, calcium crystal deposition in joints is common and remains asymptomatic in most adults, and it is, therefore, difficult to directly link CPPD formation to the symptoms of pseudogout (74). In model systems with synthetic crystals, CPPD does stimulate strong inflammatory activation. Therefore, the prevailing proposal is that CPPD formation is an essential first step for the eventual development of the acute “gout” like symptoms (75). Signaling-wise, CPPD and basic calcium phosphate can stimulate nitric oxide and collagenase production in chondrocytes *in vitro* (76, 77). Martinon et al. found that CPPD is a strong inducer of NLRP3 inflammasome activation *in vitro* (6). In addition, CPPD crystals can inhibit neutrophil apoptosis *via* Bcl-2 (78). It is very likely that all these factors work in sync to generate the inflammatory responses to calcium salt crystals.

### Cholesterol Crystals

Cholesterol clustering *in vivo* is primarily in two forms. Gallstones (cholelithiasis) are large solid structures mostly made of cholesterol in biliary duct and gallbladder. They are results of liver cholesterol accumulation, often with a genetic disposition (79). The presence of CC in atherosclerotic lesions reflects the imbalance of cholesterol homeostasis and has by far the highest impact in human health, being one of the root causes of cardiovascular disease (CVD, a third of mortality in the developed countries). *Via* the mevalonate pathway, all mammalian cells are capable of cholesterol synthesis (80). Its metabolism is mainly in the liver in the form of biliary secretion of surplus cholesterol and bile acid. Therefore, the cholesterol transport becomes the critical regulation of its level. For the cardiovascular system, low density lipoprotein *via* its receptor transports esterified cholesterol to artery walls while high density lipoprotein mediates the reverse transport back to the liver (81). In the periphery, deposited esterified cholesterol can be converted by ester hydrolases into free cholesterol, leading to CC formation (82). In advanced CVD, accumulated CC in the plaques expand in volume and cause the rupture of the fibrous cap, leading to acute thrombosis, embolism, and clinical CVD symptoms (83, 84). More in depth analyses indicate that CC may be the culprit of the initial atherosclerotic change at the very beginning. It was found by some that cholesterol-lowering treatment was only beneficial when used early in mouse life (85). Because of their small sizes, optical imaging in tissues has not been easy. The limitation is being gradually overcome with new preparation protocols (86, 87) and imaging tools such as Raman scattering microscopy (88). A recent paper reported that endothelial cells produce CC rather quickly under cholesterol overload. The crystal deposition under endothelial cells was found as early as 1 week after feeding *Ldlr*^−/−^ mice with high fat diet (89). Those improved detections strongly suggest their involvement in much of the initial atherosclerotic development. Local accumulation of CC has been recognized as an inflammatory event (90). CC can activate the complement system *in vitro* (91, 92) as well as IL-1α production *in vivo* and *in vitro* (93, 94). The central interest is undoubtedly focused on the involvement of NLRP3 inflammasome. Some reports suggested that NLRP3 inflammasome and its components were essential for the plaque formation (86, 95) while others failed to make this observation (93, 96).

### Hemozoin

Malaria is a major cause of mortality in developing countries. In its life cycle, *Plasmodiu*m invades red blood cells and uses hemoglobin as its energy source. The product of this digestion, heme, forms hemozoin crystals (97). While *Plasmodium* itself can directly modulate endothelial permeability and cause circulation blockage (98, 99), hemozoin is a major activator of innate immunity (100), both leading to various degrees of mouse hepatocyte dysfunction *in vivo* (101). The surface of hemozoin crystals has been found to be highly active in mediating oxidative responses (102). Another property of hemozoin is its extensive phagocytosis by phagocytes in the circulation and in vital organs (liver, brain, etc.), particularly after RBC rupture (103). This phagocytic event is believed to be immune regulatory and the intensity of phagocytosis is an indicator of the disease severity (104). Macrophages and monocytes stimulated with hemozoin produce large amounts of cytokines *in vitro*, including TNFα, MIP1α, and β (105), chemotactic factors (106), ROS and nitric oxide (100, 107). A large panel of signaling molecules, including ERK1/2, JAK2/STAT-1, NFkb, and Syk kinases were all reportedly involved (108). The exact recognition mechanism is not clear, although in one report TLR9 was involved (109). This finding is controversial as others have suggested the contamination of *Plasmodium* DNA in the isolated hemozoin (110–112). By contrast, the activation mediated by NLRP3 is well characterized and has been supported by multiple research groups. Shio et al. found that hemozoin-induced NLRP3 inflammation and IL-1β production were downstream of Syk kinase. Importantly, deficiencies in NLRP3 components protected the host from one strain of malaria, *Plasmodium chabaudi adami* (113). Another paper around the same time suggested that hemozoin mediated inflammatory responses *in vitro* and *in vivo*, particularly NLRP3 activation, was *via* induction of uric acid release (110).

## Particulate Adjuvants

Adjuvants are used to increase host responses to otherwise low immunogenic antigens. They can be roughly divided into delivery tools and immune potentiators (12, 114). Serving both purposes, particulate adjuvants are a subcategory of immune enhancers and are the first preparation used in human vaccine. Glenny’s work has been sufficiently discussed in vaccine reviews (115). However, one particular point worth noting is that while the early workers Glenny and Maschmann et al. used alum in various chemical composition to precipitate and stabilize diphtheria toxin, they did not recognize the immune stimulatory effects of this crystalline structure (116–118). In the last two decades, our understanding of particulate adjuvants has seen some dramatic revisions.

Alum usually refers to trivalent inorganic aluminum salts, including Al(OH)_3_ and AlPO_4_ (119, 120). For decades, it was thought that alum served as an *in vivo* depot for the associated antigens, prolonging antigen availability. This notion was proven incorrect by several experiments (121–123). In 2004, it was reported that injection of alum resulted in the accumulation of IL-4-producing monocytic Gr1^+^ cells in the spleen (124). Till date, it is still not clear how this population mediates immune response, although it is involved in TH1/TH2 bias (125). In 2008, Eisenbarth et al. reported a deficiency in multiple antibody subtype production in response to alum in NLRP3^−/−^ mice (126) suggesting that alum’s immunogenicity may be related to its ability to activate NLRP3 inflammasome. A follow-up paper using similar mouse models by Tschopp’s group, however, reported a reduction in IgE production only (127). In other reports, including from our own group, NLRP3/Caspase-1 axis was not found to be essential for alum-mediated antibody production (128–130). Later on, two groups reported that DNA release triggered by local alum injection might be responsible for its adjuvanticity (131, 132). Ishii’s group further suggested that in the process, alum triggered activation of TBK1/IRF3, leading to IgE isotype switching (132). Whether this signaling event requires STING, a sensor for intracellular DNA is still a topic of discussion (131). In addition, a recent paper suggested that commercially available DNase may have proteolytic activities, which at least partially explained the reduced adjuvanticity following DNA removal in alum-treated mice (133). Therefore, the role of DNA release in alum’s adjuvant effect still requires more carefully controlled analyses. Our group proposed an alternative mechanism. Using atomic force microscopy, we found that alum crystals bound to DC plasma membrane lipids and triggered an abortive phagocytic response. DCs thus activated showed enhanced binding to CD4^+^ T cells *via* ICAM-1 and LFA-1 (128), leading to better T cell priming by DCs.

The notion that uric acid being a particulate immune adjuvant was not derived from its ability to trigger gouty inflammation. It was found that dead cells had strong adjuvant effect when delivered with protein antigens; the active fraction was molecularly identified by chromatography and mass spec analysis (134). It was found that soluble uric acid did not have any adjuvant effect, yet it turned stimulatory upon crystallization. It has been difficult to visualize MSU deposition *in vivo* following immunization because the microcrystals were not compatible with imaging preparation protocols. Although the ability of uric acid to serve as an adjuvant is confirmed (135) in recent years, MSU has been gradually recognized as a “cryptic” adjuvant, in that many immune responses appear to require background uric acid. We have reported that MSU crystallization requires endogenous antibodies that stabilized the initial crystal formation. In the absence of antibody as in the IgH mice, uric acid did not serve as an endogenous adjuvant (58). Several reports have found that removal of uric acid *in vivo* significantly reduced airway inflammation (136) and immune responses to antigens released from dead cells (2, 137). In addition, other groups have found that uric acid may be the conduit for the immune stimulatory effect of hemozoin and alum adjuvant (110, 138).

Virus-like particles (VLPs) are a relatively new technology and originated from the vaccine preparation with killed pathogens. They are assembled viral proteins with a resulting morphology similar to original viruses. These particles are highly stimulatory in comparison with their soluble proteins and at the same time free from the safety concerns associated with attenuated virions. With recombinant technologies, bacterial, viral, mammalian, and other expression systems have been used to successfully produce VLPs. Although VLPs can be considered adjuvants in comparison with free proteins, their efficacy can be further enhanced by other adjuvants (139). The mechanistic basis for the enhanced immunity is a concept termed geometric pathogen-associated structural pattern (140). In general, all VLPs form unique repetitive surface structures (140, 141). As these patterns bare the signature of invading pathogens, they are potent in activating antigen presenting cells and mediate efficient migration of these cells to draining lymph nodes. They are also able to bind to naturally existing antibodies and fix complement (142), further enhancing their immune stimulation.

Nanoparticle adjuvants were a product of time that started two decades ago. The term defines the size but posits no limitation on its chemical/structural details. One of the most frequently tested is polylactic-co-glycolic acid and polylactic acid, for their biodegradability and easy incorporation of antigens and drugs (143). They are known to induce antibody titers similar to those adjuvanted regimens. Other popular selections are liposome and micelle-based preparations. Overall, nanoparticles can easily enter solid tumors (144). This is likely the result of extensive pathways used for the uptake of these particles, including pinocytosis, and clathrin and caveolin-dependent endocytosis (145). These particles have been used as an efficient tool for delivery mainly due to their protection (146) and controlled release (147, 148) of associated antigens. Similar to other particulate antigens, nanoparticles can trigger cross-presentation and CD8^+^ T cell responses (149), a feature sought in viral vaccine and tumor immune therapy.

## Solid Particle-Induced Host Cell Responses

### Inflammasome

Solid/amorphous/crystalline/fibrous structure-mediated cellular responses are a major part of inflammasome research, particularly signaling associated with NLRP3 inflammasome. In 2006, Tschopp’s group reported that MSU and CPPD-induced IL-1β production was dependent on NLRP3 inflammasome components, NLRP3, ASC, and caspase-1 (6). This landmark experiment started the intense pursuit of inflammatory mechanisms associated with solid particles. Subsequently, a series of papers described the requirement for NLRP3 in IL-1β production in response to silica, asbestos, and metal oxides. With limited exceptions, it is reasonable to assume that the bulk of inflammation associated with solid structures is dependent on NLRP3 inflammasome. However, the molecular events that lead to NLRP3 activation are still being debated. Several intermediate conduits have been proposed including ROS production, lysosome rupture, K^+^ efflux, and Ca^2+^ influx (150, 151).

### Reactive Oxygen Species

**Figure 1:**
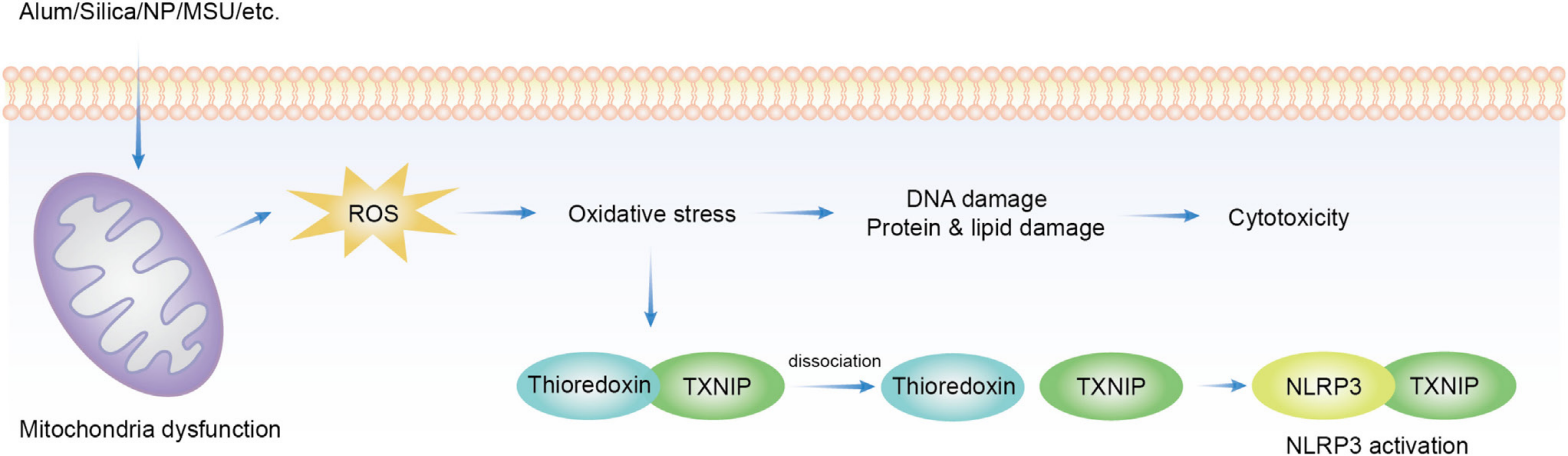
Overview of reactive oxygen species (ROS) implicated in solid particle-induced cell death and NACHT, LRR, PYD domains-containing protein 3 (NLRP3) inflammasome activation. Solid particle treatment causes mitochondrial stress and ROS production. The oxidative stress mediates the damage of DNA, proteins, and lipids, which may be an important cause of cytotoxicity. ROS production also induces NLRP3 inflammasome activation by dissociating thioredoxin-interacting protein (TXNIP) from thioredoxin then allows its binding to NLRP3, which leads to NLRP3 activation.

In the process of ATP production in mitochondria, oxygen is ideally reduced to water. However, when this process is not complete, ${\text{O}}_{2}^{-}$ escaped from this pathway becomes the source of a series of oxidizing chemicals, including hydrogen peroxide and hydroxyl radicals (152), collectively termed as ROS. These products become a part of cellular signaling network–redox biology. ROS signaling is also essential for both innate and adaptive immunity (153, 154). The excess of this production leads to oxidative stress, which is at the core of cellular aging and degenerative diseases such as sclerosis and neoplasm. ROS production can be readily induced by solid structures of various sizes and shapes, and to some extent of distinct chemical compositions. Many nanoparticles, copper, iron, cerium, zinc, nickel, titanium, aluminum oxides, gold, silver (155), silica (156), MSU (157), asbestos (158), and alum were found to induce ROS. ROS blockade with ROS scavenger or inhibition of nicotinamide adenine dinucleotide phosphate oxidase (NADPH) oxidase suppressed NLRP3 activation induced by MSU, asbestos (159), silica (158), and hemozoin (160). In the process, the conduit was proposed to be thioredoxin-interacting protein (TXNIP). TXNIP dissociates from thioredoxin in a ROS-sensitive manner and then binds to NLRP3 leading to its activation (157) (Figure 1). This proposal has not been completely satisfactory. First, a lot of stimuli induce ROS production but NLRP3 activation is not common to all of them, i.e., cytochrome P-450 oxidase uncoupling, xanthine oxidase activation, mitochondrial respiration, and various peroxisome oxidase activations (161). Inflammasome activation was not increased but suppressed in enhanced production of ROS in superoxide dismutase-1-deficient macrophages (162) while NADPH oxidase deficiency boosted the activation (163). Second, the source of ROS responsible for NLRP3 inflammasome activation remains unclear. The inhibition of mitochondrial complex I and II did not reduce asbestos-induced NLRP3 activation *in vitro* (159), arguing against mitochondria as the origin of ROS.

### Lysosomal Rupture

Another leading proposal for crystal-mediated NLRP3 inflammasome activation is *via* lysosome rupture. In this model, phagocytosis of solid particles triggers lysosome destabilization and release of cathepsin B, which activates NLRP3. Lysosome rupture blockage and cathepsin B inhibitor reduced NLRP3 activation in mouse macrophages *in vitro* induced by alum (164) and silica (165). Artificial induction of lysosome rupture with L-leucyl-L-leucine methyl ester (LLOMe) also led to NLRP3 activation that was blocked by cathepsin B inhibitor (166). However, this notion is controversial. In some reports, cathepsin B deficiency failed to reduce IL-1β production *in vitro* stimulated by MSU and silica (151, 167) or hemozoin (160). A possible explanation for this contradiction is the off-target effect of cathepsin B inhibitor as it was found to block NLRP1b inflammasome (168). Another explanation is that multiple cathepsins are involved in NLRP3 activation as the cathepsin family is highly conserved and cathepsin B inhibitor, Ca074Me, was found active toward other members (169). Furthermore, the authors found that not only NLRP3 activation but also particle-induced cell death was dependent on multiple redundant cathepsins (169, 170).

### K^+^ Efflux

In recent years, the role of K^+^ efflux in NLRP3 activation has become the center of attention. High extracellular K^+^ was reported to inhibit almost all known NLRP3 agonists *in vitro*, including hemozoin (160), silica and asbestos (159), MSU, Nigericin, and ATP (171), and bacterial pore-forming toxin, alum, CPPD, and LLOMe (151). Glyburide, a K^+^ channel blocker, also inhibited NLRP3 inflammasome *in vitro* (172), which appears to confirm the role of K^+^ efflux. The intracellular sensor for the reduced K^+^ and how it is linked to NLRP3 activation are not clear. The idea of K^+^ efflux as the upstream signal of NLRP3 came from the understanding that extracellular K^+^ blocks intracellular K^+^ outward motion during a typical cycle of eukaryotic membrane depolarization/repolarization (171) and assumed that particulate substances triggered a sustained drop of intracellular K^+^. However, experimental high K^+^ depolarizes the membrane and reduces the membrane potential (typical −40 to −80 mV) to near neutrality, and the membrane potential is a critical parameter for much of the eukaryotic biology (173–177). We recently found that both membrane depolarization and hyperpolarization were sufficient to block NLRP3 inflammasome activation without involving large amounts of K^+^ moving across the plasma membrane (our own observations). Therefore, molecular details of K^+^ efflux and NLRP3 inflammasome activation need to be further scrutinized. In addition to K^+^, Ca^2+^ influx is induced by numerous NLRP3 activators (178). Ca^2+^ influx was suggested to be important for NLRP3 activation since thapsigargin, an inhibitor of the sarcoplasmic/ER Ca^2+^-ATPase, incubation in Ca^2+^ free media (179), or BAPTA-AM (intracellular Ca^2+^ chelator) (180) significantly suppressed NLRP3 inflammasome activation in ATP-stimulated BMDM. Another piece of evidence was the calcium-sensing receptor activation stimulated NLRP3 inflammasome and knockdown of the receptor had the opposite effect (180). However, there are reports arguing against the Ca^2+^ influx model. One group found that extracellular Ca^2+^ activated NLRP3 through K^+^ efflux (151). Another group, on the other hand, suggested that Ca^2+^ influx was neither necessary nor sufficient for NLRP3 activation during ATP, Nigericin, and LLOMe stimulation (181). In a report aiming to bridge the two ion-dependent models, K^+^ and Ca^2+^ visualization sensors were used and the results suggested that K^+^ efflux was necessary for sustained Ca^2+^ influx while K^+^ efflux was independent of Ca^2+^ influx (182).

### Cell Death

**Figure 2:**
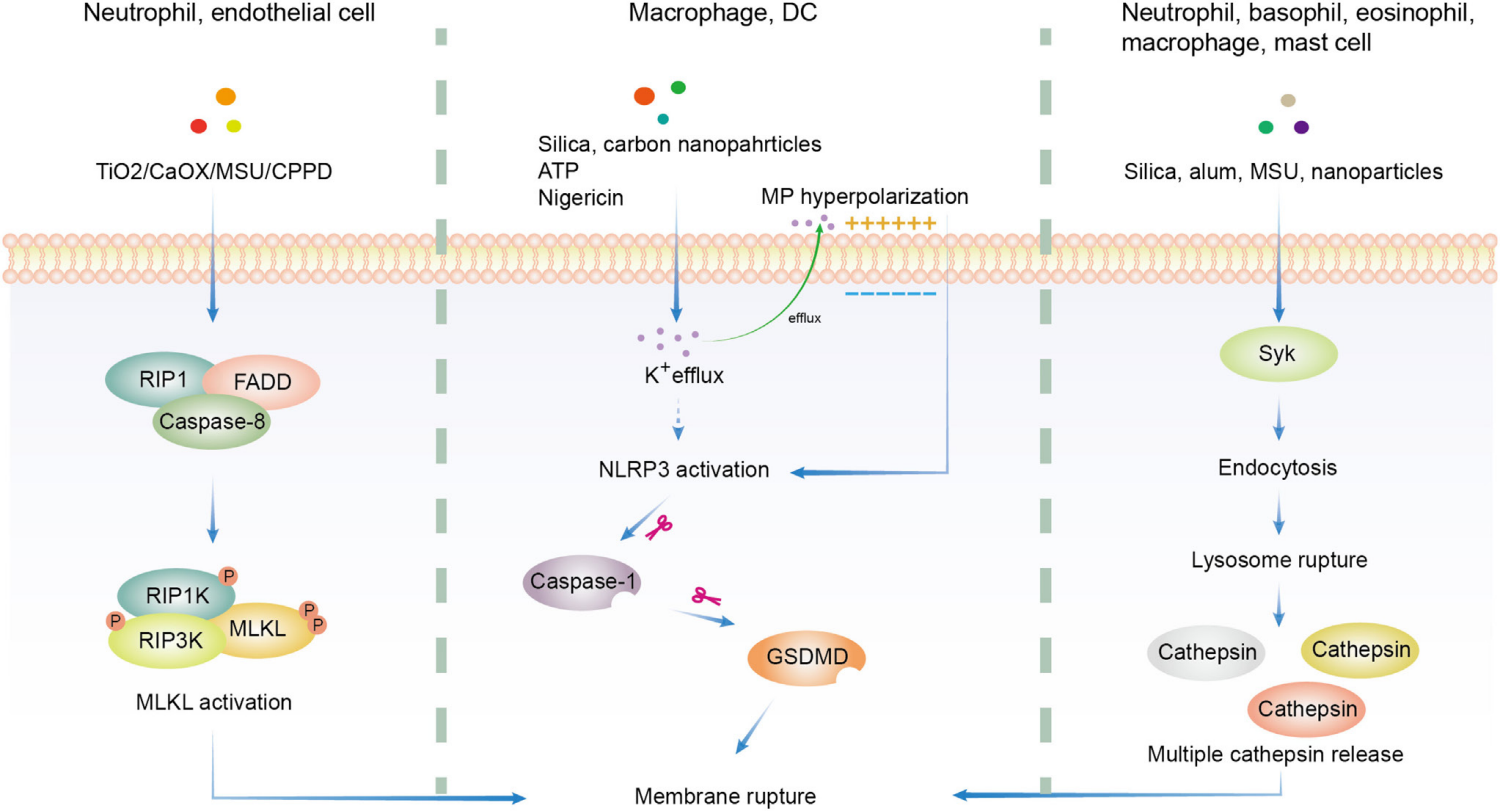
Overview of three major hypotheses of solid particle-induced cell death. In addition to cell death related to reactive oxygen species production, there are three major proposals on solid particle-induced cell death. The first is through receptor-interacting serine/threonine-protein kinase 1/3 (RIPK1/RIPK3)-mixed lineage kinase domain-like (MLKL)-driven necroptosis, which was found in calcium oxalate, monosodium urate (MSU), calcium pyrophosphate dihydrate (CPPD), cystine, TiO_2_, and calcium phosphate-treated epithelial cells and neutrophils. The second is through K^+^ efflux-activated NACHT, LRR, PYD domains-containing protein 3 (NLRP3)-dependent pyroptosis, which was found in silica, asbestos, carbon nanoparticles-treated macrophages and dendritic cells (DCs). The third is through lysosome rupture-released multiple redundant cathepsins after Syk-dependent phagocytosis, which was found in silica, alum, MSU, and nanoparticle-treated neutrophils, basophils, eosinophils, macrophages, and mast cells. GSDMD, gasdermin D.

Many particles with different chemical composition, morphology, size, hydrophobicity, and ionic charge were proved to be cytotoxic. Thus a lot of efforts were made to found a common pathway. Generally, solid particle-induced cell death relies on cellular uptake, indicating that phagosome or lysosome may be important in this type of cell death (165, 183, 184). Downstream of particle phagocytosis is the lysosome rupture and ROS production, which gives rise to oxidative stress (185–187). Following oxidative stress are mitochondrial dysfunction, DNA damage, and protein/lipid oxidation (185, 188). These factors work together to induce the eventual cytotoxicity (155). Several additional pathways have been proposed in the literature (Figure 2).

Silica-containing particles are ubiquitous, found in volcanic ashes (189), materials made from quartz and kaolin (190), and dusts (34). Silica dust (nano and crystalline particles) is an environmental and occupational hazard, as observed in construction, mining, ceramics, and foundries industry. This topic has been discussed in several reviews and very well documented clinically (191, 192). Silica has been found to be cytotoxic for a long time and believed to be responsible for silicosis (158, 159), and contribute to several types of cancer, infection (such as TB and Salmonella) (193–195) and autoimmune diseases (196). As we discussed earlier, crystalline silica is more potent in inducing alveolar macrophage death than colloidal and amorphous counterparts (34). Signaling-wise silica induced NLRP3 activation in LPS-primed macrophages *in vitro* (159). Some reports supported the notion that silica induced NLRP3-dependent pyroptosis, which relied on K^+^ efflux and caspase-1 activation. However, others argued against the dependence on NLRP3 (151, 158, 170). A report found the reliance on receptor-interacting serine/threonine-protein kinase 3 (RIPK3)-mixed lineage kinase domain-like (MLKL)-driven necroptosis (9) while another group suggested the importance of redundant cathepsins (170). Furthermore, some groups reported that silica promoted cell death *via* apoptosis through mitochondria damage pathway initiated by oxidative stress (156, 197). A slight variation was the proposal suggesting that silica induced both apoptosis and necrosis that depended on the transmembrane potential change of mitochondria. Hyperpolarization induced caspase-3 and 9-mediated apoptosis while depolarization induced no caspase activation during necrosis (198). In addition, using ATP synthesis inhibitors, oligomycin and 2-deoxyglucose, it was observed that decreased ATP level induced NLRP3 activation and necrosis while increased ATP led to apoptosis (199, 200). Therefore, crystal-induced cell death may be also under the control of ATP levels. The extreme redundancy in the types of cell death is difficult to comprehend, likely resulting from the different system setups.

Similar to silica, alum has been reported to induce lysosome rupture thus activating NLRP3 inflammasome (126, 127, 165, 201, 202). Cell death, however, was not determined in the majority of those papers except two reported that NLRP3 and caspase-1 deficiencies did not affect alum-induced macrophage cell death (126, 151). On the other hand, aluminum oxide nanoparticles were reported to depolarize cell membrane and lead to significant cell death in epithelial cells (203). Lima et al. reported that alum-induced macrophage cell death *in vitro* was a direct consequence of lysosomal membrane rupture without involving NLRP3 signaling cascade (166). Although MSU has been used as a model system for NLRP3 inflammation activation, much less is known about its ability to induce cell death although there is one group found that RIPK1-RIPK3-MLKL signaling pathway may be critical for MSU and other crystals-induced neutrophil extracellular DNA release and cell death (7). MSU was found to induce neutrophil PI3K activation, downstream of Syk and Src family kinases. PI3K is a critical element regulating the degranulation of neutrophils, a mechanism contributing to the pathogenesis of gout (204). As direct membrane binding was believed to be important for MSU-induced Syk activation (69) and NLRP3 inflammasome (167), we made a Syk conditional knock out mouse model and found that Syk deficiency indeed reduced MSU-induced cell death (our own observations). The exact mechanism of how MSU activates Syk and Src pathways thus induces cell death remains unclear.

A lot of metal oxide nanoparticles exhibited cytotoxicity (40, 205, 206), as did other common nanoparticles, including CNT, Fullerene (207), dental calculus (208), asbestos (36), carbon black nanoparticles (209), and quantum dots (210). The mechanisms of cytotoxicity, however, can be quite different. Most of them were found to induce ROS production that was associated with mitochondrial dysfunction (155). Unlike other particles, quantum dot treatment increased FAS expression and membrane lipid peroxidation that led to the impairment of mitochondria in human neuroblastoma cells (210). In addition, RIPK1-RIPK3-MLKL axis was proven important in human and murine renal tubular cell death induced by TiO_2_ and calcium oxalate (8). Anders’s group reported that MSU, calcium oxalate, CPPD, and cystine crystals mediated cell death of kidney epithelial cells (9) that were blocked by necrostatin-1 (inhibitor of necroptosis). This type of cell death was independent of caspase activation, suggesting that NLRP3 inflammasome activation associated with those crystals was not responsible for their cell death (211). As the most abundant innate immune cells, neutrophils phagocytose large amounts of crystals (212). In doing so, they process a special type of cell death by releasing their own DNA to trap those particles, a program called neutrophil extracellular traps (NET), including MSU, Silica, calcium oxalate, calcium phosphate, and asbestos (7). RIPK1-RIPK3-MLKL signaling pathway was found to be critical for this programmed cell death (NETosis) (7, 213). NETosis was also observed in eosinophils and basophils upon particle contact (212).

## Concluding Remarks

Solid particle-mediated cellular responses are an old topic of medicine and becoming more diverse in modern life style. Particulate matters impact us in multiple ways. They represent the latest technologies in vaccine design and cancer therapy. However, the limitations and disadvantages of using these particles and salt crystals in the development of pharmaceuticals, drugs, bio-therapeutics have not been systematically studied. In environmental exposure studies and some bacterial and viral material-based therapeutic regimens, solid particles are seldom pure, with frequent contamination of endotoxins and microbial nucleic acids. These factors must be carefully investigated. At the other end, crystalline arthropathies remain as much as a health threat as they have throughout the time. While the research on this subject has been multifaceted and increasingly intense, particularly with regard to their signaling pathways, we are far from establishing a framework of understanding how these solid structures are perceived by our cells and whether there are a set of critical events governing their cellular activation. As the variations in the types of particles and host cells involved can be extremely diverse, much work is still ahead. Several issues should be considered with higher priority. One is the signaling events in particulate adjuvants that lead to enhanced immune activation. This is critical because these adjuvants are used in population-based vaccination and new varieties are coming into clinical tests. New mechanistic insights will certainly benefit the better designs of vaccines. Another important topic is to develop a systematic approach to study the host responses toward nano- and micro-particles. The chemical and geometrical properties of those substances have been studied for decades and their signaling events have been one of the leading topics in immunology for 20 years. Thus far, we are in possession of very few consensuses and are often puzzled by conflicting data. One possible approach is to establish a model system with definable variables, such as the work on LDH by Williams et al. (48). This type of work will gradually lead to more mechanistic insights that enable us to better harness the particles that are in contact with our cells.

## Author Contributions

YS conceptualized the review and wrote the manuscript except for the inflammasome and the cell death, which were drafted by FS. YS revised the manuscript with assistance from FS.

## Conflict of Interest Statement

The authors declare that the research was conducted in the absence of any commercial or financial relationships that could be construed as a potential conflict of interest.

## Abbreviations

Alum, trivalent aluminum salts, including ALOH and AlPO_4_; BCP, basic calcium phosphate; CASR, calcium-sensing receptor; CC, cholesterol crystal; CPPD, calcium pyrophosphate dihydrate; CVD, cardiovascular disease; DC, dendritic cell; LDH, layered double hydroxides; LDL, low density lipoprotein; HDL, high density lipoprotein; LLOMe, L-leucyl-L-leucine methyl ester; MLKL, mixed lineage kinase domain-like; MSU, monosodium uric acid crystal; NADPH, nicotinamide adenine dinucleotide phosphate oxidase; NET, neutrophil extracellular traps; NLRP3, NACHT, LRR, PYD domains-containing protein 3; RIPK1/RIPK3, receptor-interacting serine/threonine-protein kinase 1/3; ROS, reactive oxygen species; SERCA, sarcoplasmic/ER Ca^2+^-ATPase; TXNIP, thioredoxin-interacting protein; TLR, toll-like receptor; VLPs, virus-like particles.
